# Cariprazine for negative symptoms in early psychosis: a pilot study with a 6-month follow-up

**DOI:** 10.3389/fpsyt.2023.1183912

**Published:** 2023-06-22

**Authors:** Sofia Pappa, Arturas Kalniunas, Jose Maret

**Affiliations:** ^1^West London NHS Trust, London, United Kingdom; ^2^Imperial College London, London, United Kingdom; ^3^Greater Manchester Mental Health NHS Trust, Manchester, United Kingdom

**Keywords:** cariprazine, early psychosis, first-episode, negative symptoms, partial dopamine agonists

## Abstract

**Background:**

Cariprazine, a novel antipsychotic drug that is a partial agonist with preferential binding to the D3 receptor, has demonstrated efficacy in clinical trials across all symptom domains, including negative symptoms, which can occur early in the course of psychotic illness. However, evidence, to date regarding its effects in early psychosis patients with primary negative symptoms has been limited.

**Objectives:**

To evaluate the efficacy of cariprazine for negative symptoms in early psychosis patients.

**Methods:**

Demographic and clinical information of the study population were collected from the electronic records and PANSS scale administered at baseline, 3 and 6 months. Tolerability and discontinuation reasons, where applicable, were also recorded.

**Results:**

Ten patients with early psychosis (four men and six women, mean age – 25.5  years) with prominent or predominant negative symptoms were treated with cariprazine (range 1.5 – 3 mg). Three patients discontinued cariprazine within the first 3  months due to patient choice, lack of response and non-compliance, respectively. In the remaining patients, there was a significant reduction in the mean negative PANSS score from baseline to 6 months (from 26.3 to 10.6), mean total PANSS score (from 81.4 to 43.3) and in the mean positive PANSS score (from 14.4 to 9.9) which correspond to a 53.1, 41.5, and 28.5% mean score reduction.

**Conclusion:**

This pilot study suggests that cariprazine is a safe and effective treatment in early psychosis, particularly for the alleviation of negative symptoms which remains an area of unmet treatment need.

## Introduction

Schizophrenia is a complex, heterogeneous and multidimensional disorder which may include several subtypes with different neurobiological underpinnings (1, 2). Given the global burden of schizophrenic and psychotic disorders in general, it is crucial to identify early-onset psychosis patients who are at high risk of adverse outcomes, especially those with poorer premorbid adjustment and primary negative symptoms (3).

The implementation of Early Intervention in Psychosis (EIP) services has played a crucial role in delivering specialized care to patients with early psychosis. The treatment guidelines and standards of care for early psychosis in the UK are primarily based on recommendations from the National Institute for Health and Care Excellence (NICE), which focus on early detection, prompt treatment initiation, and ongoing support (4). EIP teams consist of a range of professionals, including psychiatrists, psychologists, nurses, and social workers, who work collaboratively to provide a comprehensive and integrated approach to the treatment and support of individuals with early psychosis.

Primary and enduring negative symptoms are one of the core features in schizophrenia and are associated with long-term morbidity, reduced quality of life, significant psychosocial impairment, and high levels of unemployment (5, 6). These symptoms can occur early in the course of psychotic illness, especially in the prodromal phase or before the emergence of positive symptoms, and often persist during periods of clinical stability (7, 8). In first episode of psychosis studies, negative symptoms have been associated with highly disrupted premorbid psychosocial and vocational functioning (9, 10) and poor long-term recovery outcomes, including incomplete or delayed clinical remission (11). Therefore, early and effective treatment approaches that address such symptoms are of particular importance in this vulnerable population group (12).

However, evidence-based treatment alternatives for the management of prominent or predominant negative symptoms are limited and this area remains an important unmet clinical need (13, 14). One large well-controlled clinical trial (15) has demonstrated that cariprazine, a novel partial agonist with preferential binding to the D3 receptor, can be efficacious in improving predominant negative symptoms in patients with chronic schizophrenia. Furthermore, due to its favorable tolerability profile, cariprazine has been increasingly used as monotherapy for patients with early psychosis and as a successful substitute or augmentation to other antipsychotics causing troublesome side effects (16–18). Cariprazine, due to its efficacy on both positive and negative symptoms and favorable tolerability profile, could be in fact considered as one of the first-line treatments in early psychosis.

However, the evidence around its effectiveness specifically on negative symptoms in patients with early psychosis has been thus far only limited to case reports (19–21) and case series (22). Therefore, the aim of this pilot study is to evaluate the efficacy of cariprazine for negative symptoms in early psychosis.

## Methods

This prospective pilot study was conducted in West London NHS trust, a large, urban mental health provider in the United Kingdom. An NHS trust is typically a large organizational unit within the National Health Services of England, generally serving a geographical area and/or a specialized function. The West London NHS Trust comprises comprehensive community and inpatient specialist mental health services for all age groups (including hospital and day-care facilities, Early Intervention services, Crisis teams, rehabilitation services, etc) and operates within the legal NHS framework of the NHS. The study was approved by the department for audit and naturalistic research of West London NHS Trust (project number 1775) and therefore did not require additional research ethics committee approval though the principles of the Declaration of Helsinki were respected throughout the study.

The study cohort was formed of adult patients who were (a) treated with cariprazine at the time of the study, (b) under the care of Early Intervention in Psychosis (EIP), outpatient services, (c) demonstrating adequate mental capacity, and (d) providing verbal consent. The above inclusion criteria were intentionally kept broad as it was a pragmatic study and specific exclusion criteria such as substance misuse or comorbidities were not applied. Furthermore, while written consent is generally preferred in studies involving human subjects the need for written patient consent was waived as part of the specific type of real world study approval. However, informed verbal consent was obtained from all study participants and documented in electronic records. All data was anonymised to ensure confidentiality and privacy.

Eligible patients were enrolled as part of routine clinical care at an outpatient Early Intervention Service. None of the patients were hospitalized at baseline and initiation of treatment took place in the outpatient setting. Initiation of cariprazine was subject to independent clinical prescribing decision made by the treating clinicians based on their own independent medical judgment and standard of care was unaffected. The patients were monitored by their respective clinicians and multidisciplinary teams involved in their care within the Early Intervention Service. As it was an outpatient setting, medication was self-administered by the patients. However, patients were advised that they can choose to stop and switch to an alternative medication at any time during the treatment. The titration of medication was started by the patients’ treating clinician starting with the lowest dose and adjusting as needed. There were no predetermined time limits for dose adjustment after the initiation of treatment and the decision was left to the clinician’s discretion based on the drug’s specification and the patient’s tolerability and response; therefore, the dose increasing intervals varied between patients. The tolerability and safety measures were monitored through a clinical interview by their clinicians and through self-reporting. Patients were advised to contact the service and clinical team if they experienced any side effects. There was no formal requirement to involve caregivers as part of this study and there was clinical practice as usual followed in this respect.

Demographic and clinical characteristics of the study population were collected from the electronic records. Information on previous treatments and the reasons to switch to cariprazine were also gathered, alongside information around tolerability and, where applicable, discontinuation reasons. Finally, treatment response was measured using the Positive and Negative Syndrome Scale (PANSS) at baseline, 3 and 6 months. An interval scale (23) of 1 to 7 was utilized to measure individual PANSS items. The PANSS score reduction was calculated using the per-protocol population as: (baseline - 6-month score)/baseline × 100. However, the mean percentage change was derived by considering individual changes for each patient, rather than applying the formula to the mean absolute scores at baseline and 6 months.

## Results

As shown in Table 1, a total of 10 patients with early psychosis (4 males and 6 females) were enrolled in this pilot study with a mean (SD) age of 25.5 (6.7) years. Reasons for cariprazine initiation mostly included prominent or predominant negative symptoms and/or side effects/tolerability issues (4/10) with previous antipsychotic medication. The main side effects included hyperprolactinaemia (3/10) and weight gain (1/10). Only three (30%) patients had no previous exposure to antipsychotic medication. Half of the study population (50%) were on other concomitant treatments, including one on antipsychotics (1/10) and four on antidepressants (4/10).

**Table 1:** Demographics, treatment, and tolerability.

Case	Gender	Age	Reasons for initiating CPZ	Previous medication	Concomitant medication	CPZ dose	Tolerability/discontinuation
1	Female	28	Negative Sx	Risperidone	None	3 mg	Good tolerability
2	Male	23	Negative Sx	Nil	None	1.5 mg	Good tolerability
3	Female	22	Negative Sx	Olanzapine	None	1.5 mg	Could not tolerate 3 mg
4	Male	21	Negative Sx & hyperprolactinaemia	Haloperidol	Haloperidol	1.5 mg	Good tolerability
5	Female	20	Negative Sx & SE	Quetiapine	Sertraline	3 mg	Good tolerability
6	Female	36	Negative Sx & SE	OIanzapine	Vortioxetine	3 mg	Good tolerability
7	Female	22	Positive and negative Sx	Nil	Mirtazapine	3 mg	Good tolerability
8	Male	21	Positive & Negative Sx	Nil	None	3 mg	Stopped in 34 days (poor response)
9	Female	38	Poor compliance with previous Tx	Zuclopenthixol decanoate LAI	None	3 mg	Stopped in 70 days (feeling of tightness)
10	Male	23	SE	Olanzapine	Vortioxetine	3 mg	Stopped in 72 days (non-compliance)

CPZ, cariprazine; Sx, symptoms; Dx, disorder; SE, side effects; Tx, treatment; LAI, long-acting injection.

Most patients (70%) tolerated cariprazine well without any significant concerns and adhered to treatment throughout the 6-month follow-up period. One patient (case 3) could not tolerate higher doses than 1.5 mg due to side effects. Three patients discontinued treatment after 34, 70 and 72 days due to lack of response, patient choice (experienced vague feelings of tightness), and non-compliance, respectively. Of these, the first two patients were switched to alternative antipsychotics and the third had to be hospitalized due to ongoing non-concordance with medication and relapse. The remaining patients who completed six months of treatment on cariprazine reported good tolerability at doses ranging between 1.5 mg and 3 mg. None of these patients were hospitalized at baseline or at any time during the 6-month follow-up period.

**Figure 1:**
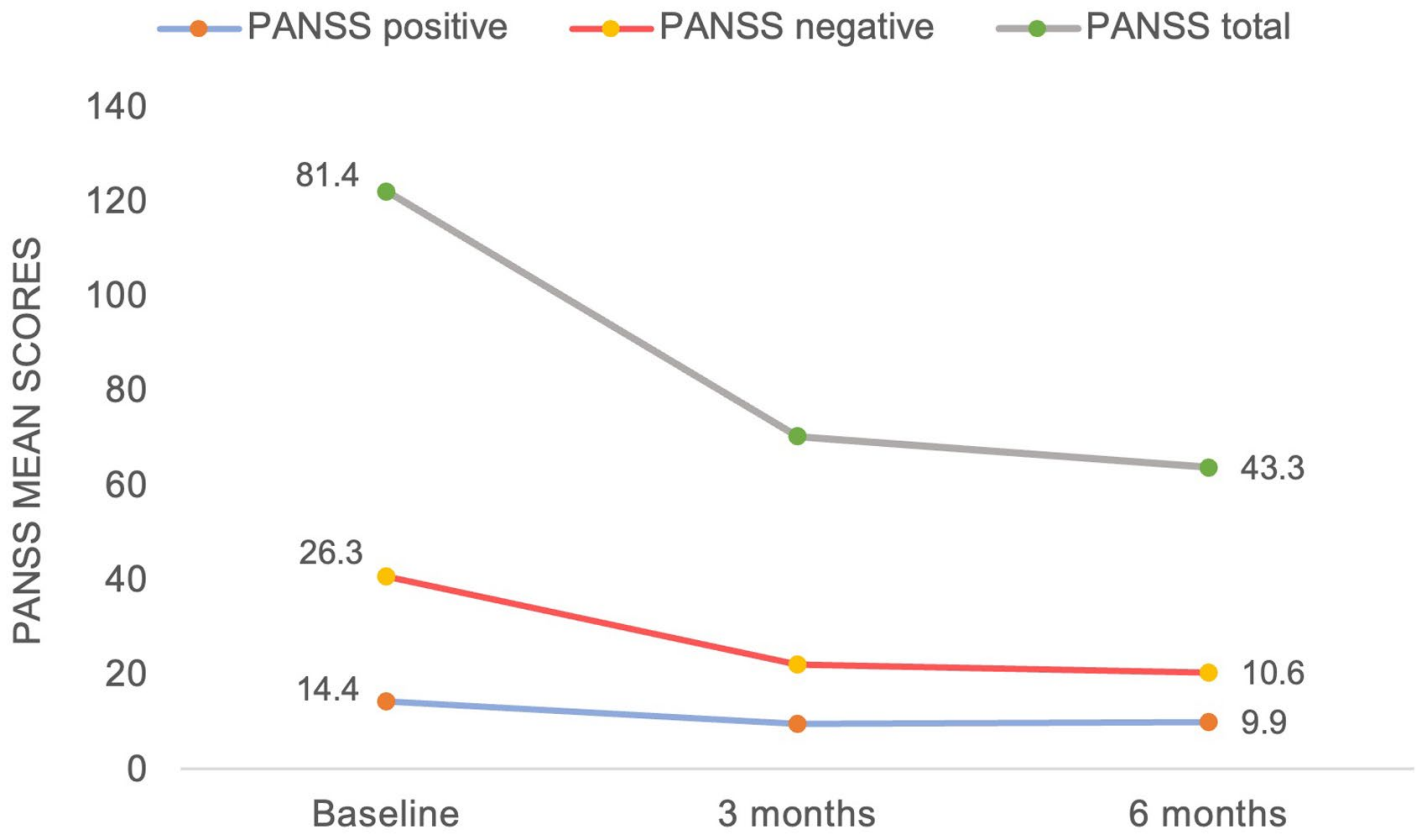
PANSS mean score change from baseline to 6  months.

**Figure 2:**
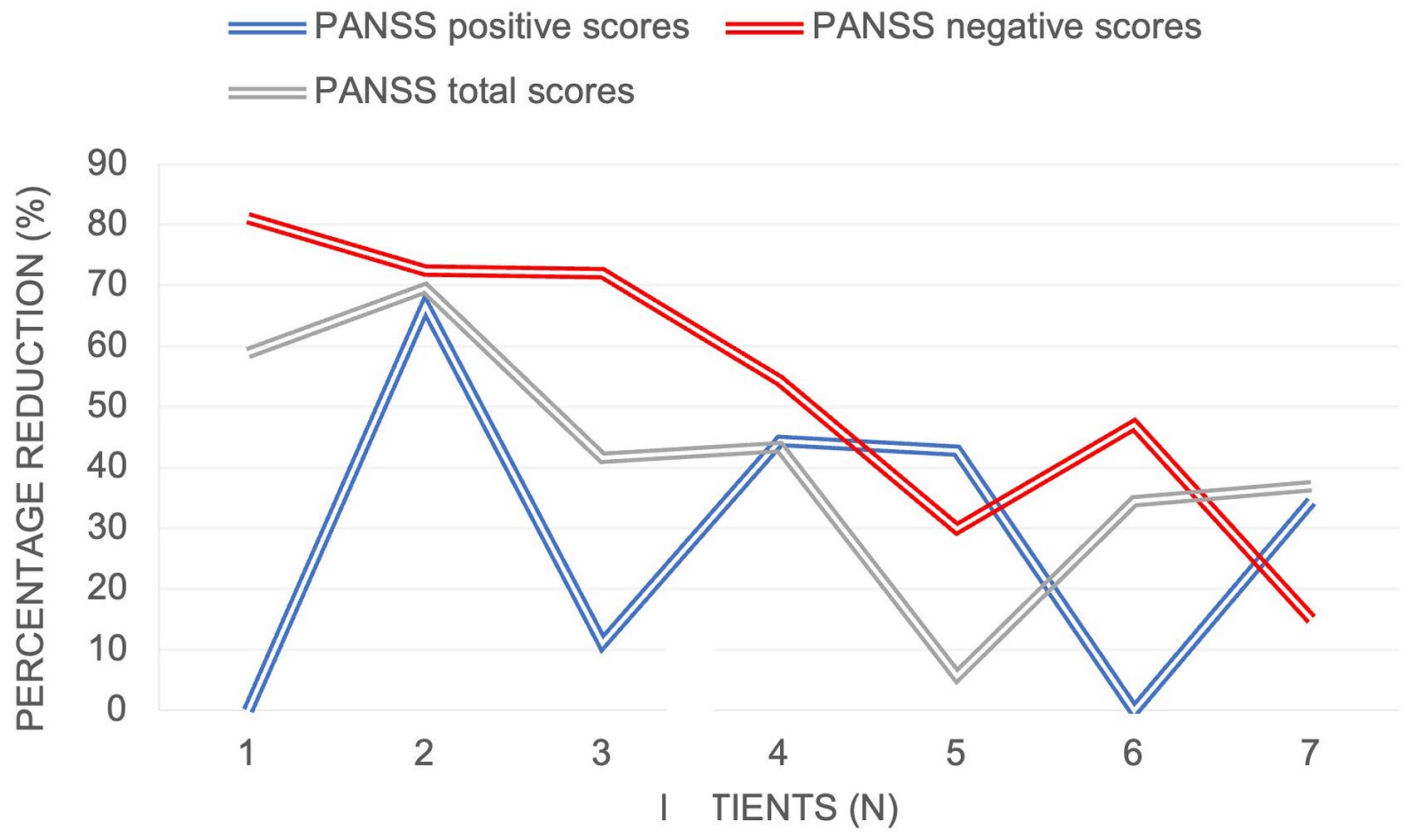
Percentage reduction in PANSS scores from baseline to 6  months.

With regards to the treatment efficacy (Figure 1), there was a significant reduction in the mean negative PANSS score from baseline to 6 months (from 26.3 to 10.6), mean total PANSS score (from 81.4 to 43.3) and in the mean positive PANSS score (from 14.4 to 9.9). As demonstrated in Figure 2, the most prominent percentage reduction in 5 out of 7 cases was across PANSS negative scores with less pronounced reductions in overall positive scores. The mean percentage decrease from baseline to 6 months in negative PANSS scores was 53.1%, total PANSS scores – 41.5% and positive PANSS scores – 28.5%.

## Discussion

To our knowledge, this is the prospective study with a 6-month follow-up to assess the effectiveness of cariprazine on negative symptoms in patients with early psychosis. Consistent with other case reports (19, 20), the results of our pilot study demonstrated a substantial decrease in PANSS scores across all symptom domains, with the most pronounced effects noted on negative symptoms. The majority of patients (6/7) had prominent or predominant negative symptoms (14), which meant that they had relatively low positive symptom scores at baseline. This could explain a more modest treatment effect on positive symptom reduction (28.5% mean score reduction) compared to other symptoms domains.

The results of this study also support the existing evidence (21, 24, 25) that cariprazine could be considered as a viable alternative to other antipsychotics associated with tolerability issues. Its unique pharmacological profile, which includes partial agonism of dopamine receptors with preferential binding to D3 over D2 receptors and low/absent affinity to histaminergic and cholinergic receptors (26, 27) has been associated with an improvement in negative symptoms (15, 28) and low rates of adverse events, including metabolic side effects (29) and hyperprolactinaemia (15, 30).

In patients with first-episode psychosis, negative symptoms have been attributed to worse functional and clinical outcomes (11, 31, 32). Furthermore, incomplete resolution of both positive and negative symptoms or poor tolerability to medication can lead to non-adherence to treatment (33), which may result in further relapses, hospitalization, and increased risk of suicide (34). Such poor outcomes could be potentially modified or mitigated by targeting early and full symptomatic remission and carefully selecting antipsychotic therapy that most suits individual patient needs. Evidence from several meta-analyses (35, 36) suggests that the efficacy differences between antipsychotics are only marginal, whereas differences in side-effects are more marked.

It is noteworthy, that considerably lower doses of cariprazine were sufficient to achieve an improvement across all symptom domains in this study compared to patients with chronic schizophrenia (15). This is in keeping with the existing notion that the treatment response is quicker and more favorable in this patient group, except for negative symptoms (37). Not dissimilar to our study, however, where a significant reduction in PANSS scores was already noted at 3 months (Figure 1), a recent case series (22) described a relatively quick treatment response with the use of cariprazine for negative symptoms (range 1–8 weeks); though important information relating to the nature, etiology and assessment of such symptoms were not specified.

Main limitations of this pilot study include its small sample size and potential confounding factors, such as, variable previous and concurrent exposure to antipsychotics or other psychotropics, presence of substance misuse and other potential psychiatric and physical comorbidities, different degree of symptom severity at baseline as well as duration of illness and untreated psychosis and lack of control for pseudo-specificity. Furthermore, the findings must be interpreted cautiously as some patients already had relatively low scores at baseline, which could have led to an underestimation of the mean percentage reduction.

## Conclusion

Findings from this small prospective study suggest that cariprazine is a safe and effective treatment in early psychosis, particularly for the alleviation of negative symptoms which remains an area of unmet treatment need. However, further research is needed, preferably involving studies with a randomized, controlled design and adequate sample size.

## Data availability statement

The raw data supporting the conclusions of this article will be made available by the authors, without undue reservation.

## Ethics statement

Ethical review and approval was not required for the study on human participants in accordance with the local legislation and institutional requirements. Written informed consent for participation was not required for this study in accordance with the national legislation and the institutional requirements.

## Author contributions

JM contributed to the acquisition of data for the work, critical revision of the article, final approval of the version to be published, agreed to be accountable for all aspects of the work. All authors contributed to the article and approved the submitted version.

## Conflict of interest

SP reports grants and honoraria outside the submitted work.

The remaining authors declare that the research was conducted in the absence of any commercial or financial relationships that could be construed as a potential conflict of interest.

## Publisher’s note

All claims expressed in this article are solely those of the authors and do not necessarily represent those of their affiliated organizations, or those of the publisher, the editors and the reviewers. Any product that may be evaluated in this article, or claim that may be made by its manufacturer, is not guaranteed or endorsed by the publisher.

## References

[1] Peralta V Cuesta MJ. Clinical models of schizophrenia: a critical approach to competing conceptions. Psychopathology (2000) 33:252–8. doi: 10.1159/000029154 10965282

[2] De Berardis D De Filippis S Masi G Vicari S Zuddas A. A neurodevelopment approach for a transitional model of early onset schizophrenia. Brain Sci. (2021) 11:275. doi: 10.3390/brainsci11020275 33672396 PMC7926620

[3] Díaz-Caneja CM Pina-Camacho L Rodríguez-Quiroga A Fraguas D Parellada M Arango C. Predictors of outcome in early-onset psychosis: a systematic review. NPJ Schizophr. (2015) 1:14005. doi: 10.1038/npjschz.2014.5, PMID: 27336027 PMC4849440

[4] National Institute for health and care excellence (NICE). Psychosis and schizophrenia in adults: Prevention and management. Clinical guideline CG178. London: NICE (2014). 31869041

[5] Buchanan RW. Persistent negative symptoms in schizophrenia: an overview. Schizophr Bull. (2007) 33:1013–22. doi: 10.1093/schbul/sbl057, PMID: 17099070 PMC2632326

[6] Rabinowitz J Levine SZ Garibaldi G Bugarski-Kirola D Berardo CG Kapur S. Negative symptoms have greater impact on functioning than positive symptoms in schizophrenia: analysis of CATIE data. Schizophr Res. (2012) 137:147–50. doi: 10.1016/j.schres.2012.01.015, PMID: 22316568

[7] Carbon M Correll CU. Thinking and acting beyond the positive: the role of the cognitive and negative symptoms in schizophrenia. CNS Spectr. (2014) 19:35–53; quiz 35–7, 53. doi: 10.1017/S1092852914000601, PMID: 25403863

[8] Mäkinen J Miettunen J Isohanni M Koponen H. Negative symptoms in schizophrenia: a review. Nord J Psychiatry. (2008) 62:334–41. doi: 10.1080/08039480801959307 18752104

[9] Best MW Grossman M Oyewumi LK Bowie CR. Examination of the positive and negative syndrome scale factor structure and longitudinal relationships with functioning in early psychosis: PANSS factor structure and functioning. Early Interv Psychiatry. (2016) 10:165–70. doi: 10.1111/eip.12190, PMID: 25277757

[10] Chang WC Lau CFC Chan SSI Hui CLM Chan SKW Lee EHM. Premorbid, clinical and cognitive correlates of primary negative symptoms in first-episode psychosis. Psychiatry Res. (2016) 242:144–9. doi: 10.1016/j.psychres.2016.05.045, PMID: 27280524

[11] Rammou A Fisher HL Johnson S Major B Rahaman N Chamberlain-Kent N. Negative symptoms in first-episode psychosis: clinical correlates and 1-year follow-up outcomes in London early intervention services. Early Interv Psychiatry. (2019) 13:443–52. Available from:. doi: 10.1111/eip.12502, PMID: 29148264

[12] Schennach-Wolff R Jäger M Mayr A Meyer S Kühn K-U Klingberg S. Predictors of response and remission in the acute treatment of first-episode schizophrenia patients--is it all about early response? Eur Neuropsychopharmacol. (2011) 21:370–8. doi: 10.1016/j.euroneuro.2010.10.003 21255982

[13] Arango C Garibaldi G Marder SR Krause M Zhu Y Huhn M. Antipsychotic drugs for patients with schizophrenia and predominant or prominent negative symptoms: a systematic review and meta-analysis. Eur Arch Psychiatry Clin Neurosci. (2018) 268:625–39. doi: 10.1007/s00406-018-0869-3 29368205

[14] Correll CU Schooler NR. Negative symptoms in schizophrenia: a review and clinical guide for recognition, assessment, and treatment. Neuropsychiatr Dis Treat. (2020) 16:519–34. doi: 10.2147/NDT.S225643, PMID: 32110026 PMC7041437

[15] Németh G Laszlovszky I Czobor P Szalai E Szatmári B Harsányi J. Cariprazine versus risperidone monotherapy for treatment of predominant negative symptoms in patients with schizophrenia: a randomised, double-blind, controlled trial. Lancet. (2017) 389:1103–13. doi: 10.1016/s0140-6736(17)30060-0, PMID: 28185672

[16] Fagiolini A Alcalá JÁ Aubel T Bienkiewicz W Bogren MMK Gago J. Treating schizophrenia with cariprazine: from clinical research to clinical practice. Real world experiences and recommendations from an international panel. Ann General Psychiatry. (2020) 19:55. doi: 10.1186/s12991-020-00305-3, PMID: 32999683 PMC7520022

[17] Correll CU Demyttenaere K Fagiolini A Hajak G Pallanti S Racagni G. Cariprazine in the management of negative symptoms of schizophrenia: state of the art and future perspectives. Future Neurol. (2020) 15:12. doi: 10.2217/fnl-2020-0012

[18] Pappa S Kalniunas A Sharma H Raza-Syed A Kamal M Larkin F. Efficacy and safety of cariprazine augmentation in patients treated with clozapine: a pilot study. Ther Adv Psychopharmacol. (2022) 12:204512532211320. doi: 10.1177/20451253221132087, PMID: 36439679 PMC9685211

[19] Bajouco M Mota D. Cariprazine on psychosis: beyond schizophrenia – a case series. Neuropsychiatr Dis Treat. (2022) 18:1351–62. doi: 10.2147/ndt.s355941, PMID: 35818373 PMC9270979

[20] Coentre R Saraiva R Sereijo C Levy P. Cariprazine use in early psychosis: three case reports. Front Psychiatry. (2021) 12:788281. doi: 10.3389/fpsyt.2021.788281, PMID: 34975583 PMC8716595

[21] Molnar MJ Jimoh IJ Zeke H Palásti Á Fedor M. Early-onset schizophrenia with predominantly negative symptoms: a case study of a drug-naive female patient treated with cariprazine. Front Pharmacol. (2020) 11:11. doi: 10.3389/fphar.2020.00477, PMID: 32390838 PMC7191004

[22] Demjaha A Iacoponi E Hansen L Peddu P McGuire P. Cariprazine as a treatment for negative psychotic symptoms in first-episode psychosis: case series. BJPsych Open. (2022) 8:e88. doi: 10.1192/bjo.2022.56, PMID: 35481438 PMC9059739

[23] Kay SR Fiszbein A Opler LA. The positive and negative syndrome scale (PANSS) for schizophrenia. Schizophr Bull. (1987) 13:261–76. doi: 10.1093/schbul/13.2.261 3616518

[24] Taube M. Case report: severe side effects following treatment with first generation antipsychotics while cariprazine leads to full recovery. Front Psych. (2021) 12:804073. doi: 10.3389/fpsyt.2021.804073, PMID: 34970176 PMC8713645

[25] Aubel T. Cariprazine: patients with treatment-resistant schizophrenia. Neuropsychiatr Dis Treat. (2021) 17:2327–32. doi: 10.2147/NDT.S315653, PMID: 34285492 PMC8286718

[26] Taylor D Chithiramohan R Grewal J Gupta A Hansen L Reynolds GP. Dopamine partial agonists: a discrete class of antipsychotics. Int J Psychiatry Clin Pract. (2022):1–13. doi: 10.1080/13651501.2022.2151473, PMID: 36495086

[27] Stahl SM. Mechanism of action of cariprazine. CNS Spectr. (2016) 21:123–7. doi: 10.1017/S1092852916000043 26956157

[28] Stahl SM. Drugs for psychosis and mood: unique actions at D3, D2, and D1 dopamine receptor subtypes. CNS Spectr. (2017) 22:375–84. doi: 10.1017/S1092852917000608, PMID: 28965530

[29] Pillinger T McCutcheon RA Vano L Mizuno Y Arumuham A Hindley G. Comparative effects of 18 antipsychotics on metabolic function in patients with schizophrenia, predictors of metabolic dysregulation, and association with psychopathology: a systematic review and network meta-analysis. Lancet Psychiatry. (2020) 7:64–77. doi: 10.1016/S2215-0366(19)30416-X, PMID: 31860457 PMC7029416

[30] Campbell RH Diduch M Gardner KN Thomas C. Review of cariprazine in management of psychiatric illness. Ment Health Clin. (2017) 7:221–9. doi: 10.9740/mhc.2017.09.221, PMID: 29955527 PMC6007710

[31] Santesteban-Echarri O Paino M Rice S González-Blanch C McGorry P Gleeson J. Predictors of functional recovery in first-episode psychosis: a systematic review and meta-analysis of longitudinal studies. Clin Psychol Rev. (2017) 58:59–75. Available from:. doi: 10.1016/j.cpr.2017.09.007, PMID: 29042139

[32] Álvarez-Jiménez M Gleeson JF Henry LP Harrigan SM Harris MG Killackey E. Road to full recovery: longitudinal relationship between symptomatic remission and psychosocial recovery in first-episode psychosis over 7.5 years. Psychol Med. (2012) 42:595–606. doi: 10.1017/S0033291711001504, PMID: 21854682

[33] Raghavan V Mohan G Gopal S Ramamurthy M Rangaswamy T. Medication adherence in first-episode psychosis and its association with psychopathology. Indian J Psychiatry. (2019) 61:342–6. doi: 10.4103/psychiatry.IndianJPsychiatry_148_17, PMID: 31391636 PMC6657546

[34] Leucht S Heres S. Epidemiology, clinical consequences, and psychosocial treatment of nonadherence in schizophrenia. J Clin Psychiatry. (2006) 67:1948–53. doi: 10.4088/JCP.v67n1216 16822090

[35] Huhn M Nikolakopoulou A Schneider-Thoma J Krause M Samara M Peter N. Comparative efficacy and tolerability of 32 oral antipsychotics for the acute treatment of adults with multi-episode schizophrenia: a systematic review and network meta-analysis. Lancet. (2019) 394:939–51. Available from:. doi: 10.1016/S0140-6736(19)31135-3, PMID: 31303314 PMC6891890

[36] Leucht S Cipriani A Spineli L Mavridis D Orey D Richter F. Comparative efficacy and tolerability of 15 antipsychotic drugs in schizophrenia: a multiple-treatments meta-analysis. Lancet. (2013) 382:951–62. doi: 10.1016/S0140-6736(13)60733-3, PMID: 23810019

[37] Zhu Y Li C Huhn M Rothe P Krause M Bighelli I. How well do patients with a first episode of schizophrenia respond to antipsychotics: a systematic review and meta-analysis. Eur Neuropsychopharmacol. (2017) 27:835–44. doi: 10.1016/j.euroneuro.2017.06.011, PMID: 28669774

